# A scoping review of antimalarial drug resistance markers in Kenya (1987–2022): toward a National Surveillance Framework and Data Repository

**DOI:** 10.1186/s12936-025-05616-y

**Published:** 2025-11-19

**Authors:** Kevin Wamae, John Magudha, Emmanuel Asiimwe, Kariuki Kimani, Regina Kandie, Kibor Keitany, Robert W. Snow, L. Isabella Ochola-Oyier

**Affiliations:** 1 KEMRI-Wellcome Trust Research Programme, Kilifi, Kenya; 2 Division of National Malaria Programme, Ministry of Health, Nairobi, Kenya; 3 Centre for Tropical Medicine and Global Health, Nuffield Department of Medicine, University of Oxford, Oxford, UK

**Keywords:** Antimalarial drug resistance, Surveillance, Database, PRISMA-ScR

## Abstract

The identification of genetic markers has revolutionized the assessment of antimalarial drug resistance. Tracking the molecular markers of resistance emerged as a valuable tool over 60 years ago, following the identification of sulfadoxine-pyrimethamine (SP) genetic resistance markers, *dhfr* and *dhps.* The Preferred Reporting Items for Systematic Reviews and Meta-Analyses extension for Scoping Reviews (PRISMA-ScR) guidelines were used. PubMed/MEDLINE, Embase, Scopus, Google Scholar and Web of Science were systematically searched to identify studies on antimalarial drug resistance markers in Kenya published in English between 01-Jan-1995 and 31-Oct-2024. The national analysis showed a regional shift in the timelines from the mutant to wild-type *crt* genotype and similarly from the mutant (CVIET) to wild-type (CVMNK) microhaplotype, with the Coast occurring earlier in 2002, while Western Kenya the change occurred later in 2008. MDR1 codons 86 and 1246, also genetic markers of chloroquine resistance have shown a full reversion to the wildtype, that was rising since 1994 in the Coast and 2003 in Western Kenya. By the time drug policy changed to SP in 1999 the *dhps* mutant genotype was already rising from 1996 in the Coast and 1998 from Western Kenya, while the *dhfr* codon 108 shift to mutant occurred as early as 1988 in the Coastal parasite populations. The World Health Organization-validated *k13* mutations were first described, P553L, in 2006 in Kisumu. This aggregation of data across Kenya demonstrates the utility of this scoping review. The compilation and standardization of over 100 studies provides a high-level, structured overview of when and where resistance markers have been surveyed. This establishes a foundational national repository to support strategic surveillance planning by the Kenya NMCP.

**Supplementary Information:**

The online version contains supplementary material available at 10.1186/s12936-025-05616-y.

## Background

Antimalarial drug resistance (AMDR) poses a major threat to malaria control efforts globally. AMDR refers to the parasites reduced susceptibility to standard antimalarial treatments, leading to prolonged or incomplete parasite clearance that can lead to treatment failure [1]. The malaria parasite has historically evolved to avoid drug action and mutations will continue to emerge to all existing and new antimalarial treatments. The identification of genetic markers of AMDR has revolutionized the assessment of this relentless malaria public health challenge [2–7]. Tracking the molecular markers of resistance emerged as a valuable tool over 60 years ago (with the identification of sulfadoxine-pyrimethamine genetic resistance markers, [3, 4]) and allowed an improved understanding of the spread and epidemiology of antimalarial drug resistance.

Chloroquine (CQ) resistance in Africa was at high levels, > 90% of the chloroquine resistance transporter (CRT) CVIET mutant haplotype present at the time of widespread clinical failures, and associated with a rise in both malaria morbidity and mortality [8, 9]. Following the cessation of CQ use, there was a decline in CVIET parasites across Africa, with some low prevalence (< 0.4%) of resistant genotypes reported in some countries, Madagascar in 2007, Malawi in 2009, Zambia in 2013 and Tanzania in 2018 [10]. Conversely, countries that have retained CQ for the treatment of vivax malaria (e.g. Ethiopia) continue to have high levels of CVIET mutations [11].

Sulfadoxine-pyrimethamine (SP), that almost universally replaced CQ as a first-line treatment in the early 2000s across Africa, has shown variable geographic emergence and rates of increase of different mutations to both dihydrofolate reductase (*dhfr*) and dihydropteroate synthase (*dhps*) genotypes [12]. While withdrawn as a treatment, SP continues to be used for the prevention of malaria during pregnancy and infancy and in combination for seasonal malaria chemoprevention. This sustained drug pressure has led to a continued rise in the quintuple ‘super resistant’ *dhfr* and *dhps* combination mutant in East Africa [13], while in West Africa, the quadruple mutant is dominant and the *dhps* 540E mutation is less frequent [14].

The World Health Organization (WHO)-validated artemisinin resistance *kelch 13* (*k13*) mutations have been detected initially in Southeast Asia [15, 16] and since 2015 in East Africa [17]. The initial and widespread, but non-significant mutation A578S (confirmed as not conferring resistance to artemisinin [7]) was described, similarly K189S found outside the propeller domain was also observed in Africa [18]. The current emerging trend of important *k13* mutations in Africa, particularly from the East and Horn of Africa are C469Y/F, R539T, P553L, R561H, R622I and A675V validated artemisinin resistance mutations and P441L and S552C as associated with resistance [19].

National antimalaria treatment policy dialogues were focused on data from studies of clinical failure during the CQ era. This began to change during the SP era where data on treatment failures was augmented with data from studies of the early identification of *Pfdhfr* and *Pfdhps* mutations [20]. Furthermore, WHO advice on using SP as an intermittent presumptive treatment for prevention is guided by data on mutation rates [21]. International and national antimalarial future drug policy discussions are now driven by molecular surveillance, where the genetic marker of resistance is known, the detection of *Pfk13* mutations, and linked to their clinical association with delayed parasite clearance [22].

The power of genetic markers of AMDR in tracking changes in malaria parasite susceptibility to widely used drugs over time and space has raised the significance of establishing malaria molecular surveillance (MMS) [23]. However, how these enhanced surveillance networks are connected within countries, between countries, data are assembled/shared/standardized and how information are presented in policy forums to guide national decision-making remains under-developed.

MMS in Africa has been reliant on national and international academic research groups with little coordinated national level surveillance or systematic, standardized approaches within countries or regionally. Importantly, data generated at national levels are not always accessible in an easy to understand format to inform action to those charged with making health policy. To address this, a scoping review of AMDR mutations in Kenya was undertaken to assemble historical and contemporary data on mutations to previously and currently used antimalarial drugs. The intention being to provide a unique MMS database that can be used by the national malaria control programme (NMCP) to better understand the past, present and possible future of parasite mutations and initiate better future coordination and submission of data assembled by research partners.

## Methods

### Country context

**Fig. 1:**
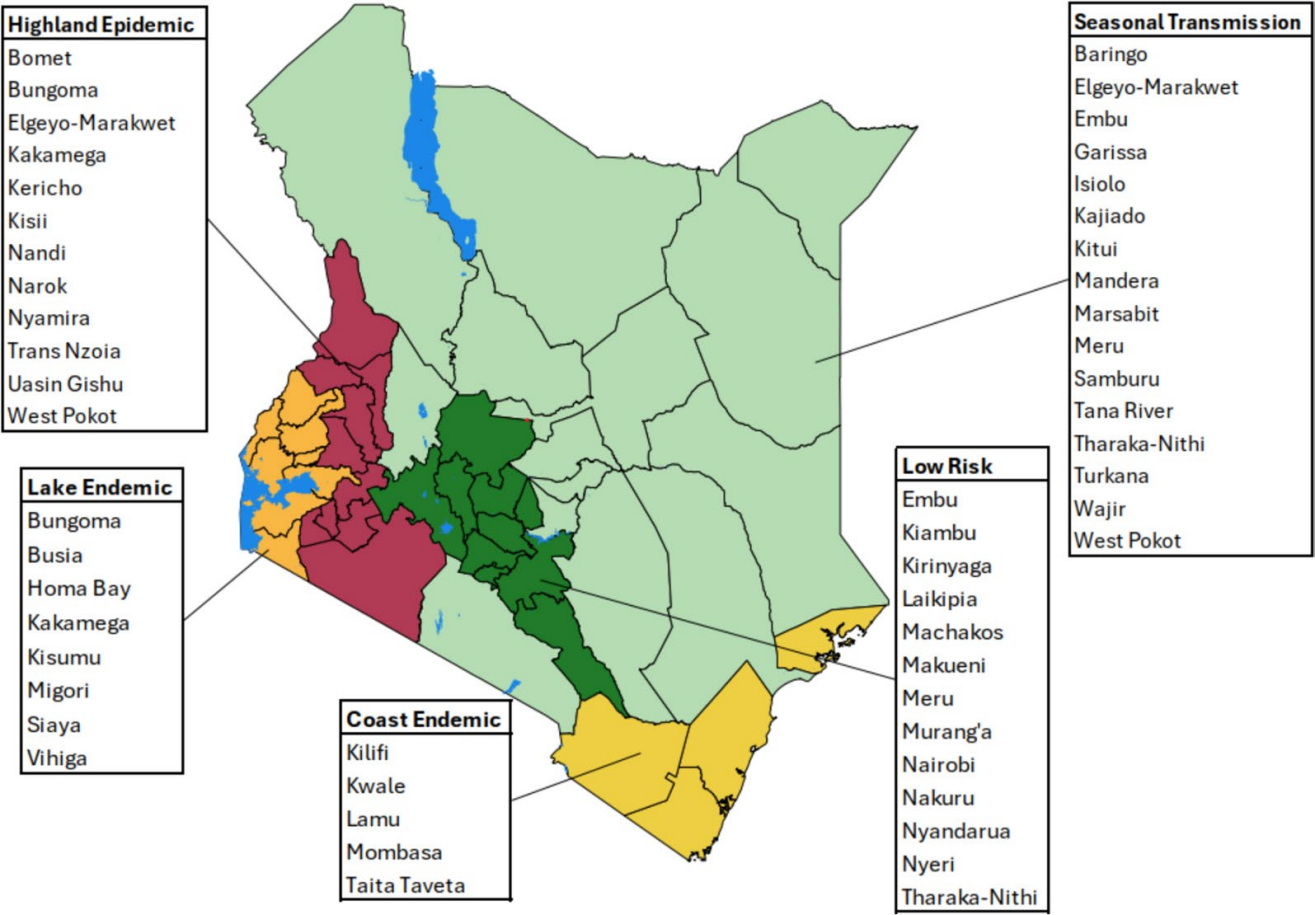
Geographic distribution of malaria epidemiological zones in Kenya. Kenya’s malaria epidemiological zones are defined by the Kenya National Malaria Control Programme. The zones reflect varying transmission patterns: lake endemic (orange) experiences high, stable transmission; coast endemic (yellow) has low to moderate year-round risk; highland epidemic (maroon) has unstable seasonal outbreaks; semi-arid seasonal (green) sees short, acute outbreaks; and low-risk areas (light green) have minimal transmission. Major lakes are in blue, with grouped county labels for each zone (Adapted from [55])

Kenya has a diverse malaria ecology ranging from areas unable to sustain transmission due to altitude/temperature limits on sporogony or semi-arid deserts that cannot support mosquito survival to intense perennial transmission. Traditionally, the most intense transmission has occurred along Kenya’s coastline and around Lake Victoria [24, 25]. Despite significant gains in the control of malaria since the launch of Roll Back Malaria nationwide, progress has been less dramatic among lakeside counties in Western Kenya and the southern coastline [24, 25]. Since 2010, the Kenyan NMCP has sub-nationally classified the country into five malaria epidemiological zones [26]. These are the lake endemic area-localities around Lake Victoria with stable, high transmission; the coast endemic area-localities along the Indian Ocean coast with low to moderate transmission; the Western highland epidemic prone areas with unstable, year-to-year fluctuations in transmission; the semi-arid seasonal area-northern, eastern, and south-eastern settings with short, acute unpredictable transmission seasons; and the very low-risk area-central highlands and Nairobi, where transmission is either low or absent (Fig. 1) [26]. This sub-national stratification has guided the delivery of malaria interventions since 2010, including vector control, early vaccine introduction and the use of intermittent presumptive treatment of malaria in pregnancy with SP, where efforts have been intensified in counties located in the first two endemicity classes around Lake Victoria and along the Indian Ocean coast [26]. Early detection, diagnosis and treatment is an important pillar of the malaria strategy, irrespective of endemic classification.

CQ resistance was first reported in Kenya in a tourist in 1978 [27] and in a semi-immune Kenyan in 1982 [28]. Thereafter, escalating treatment failures were documented for over 10 years and the Kenyan Ministry of Health, like many other African countries, were slow to respond [29]. These delays were, in part a result of poor dialogue between local research communities and policy makers [29]. Between 1997 and 1998, CQ was formally replaced as a first-line treatment with SP [30], a decision facilitated by a newly established network of researchers and malaria divisions within the ministries of health in the East Africa region, East African Network for Monitoring Antimalarial Treatment (EANMAT) [31]. This co-created network provided a platform based on mutual trust, ownership of evidence and data sharing across borders that fed directly into policy discussions. EANMAT was subsequently instrumental in providing the evidence to abandon SP in favour of artemisinin-based combination therapy (ACT) across the subregion [30, 32]. In 2004, Kenya adopted artemether-lumefantrine (AL) as first-line treatment for uncomplicated malaria, which was not implemented effectively until 2006 [30]. Subsequent drug therapeutic and molecular monitoring efforts, mainly supported by the President's Malaria Initiative (PMI) and the Centres for Disease Control and Prevention (CDC) [33], have been fragmented and reliant on independent research groups, resulting in significant gaps in coordinated national-level surveillance. Hence the need to identify, by means of a scoping review, data that has been collected during and post the EANMAT era.

### Search strategy

The review adheres to the guidelines established by the Preferred Reporting Items for Systematic Reviews and Meta-Analyses extension for Scoping Reviews (PRISMA-ScR) [34]. PubMed/MEDLINE, Embase, Scopus, Google Scholar and Web of Science were systematically searched to identify studies on antimalarial drug resistance markers in Kenya published in English between 01-Jan-1995 and 31-Oct-2024. The search strategy was tailored for each database using a combination of terms and free-text keywords related to Kenya, malaria, drug resistance, and parasite genotyping. Date restrictions and language filters were included where applicable. The complete list of search terms and syntax used for each database is presented in Supplementary Table 1.

### Inclusion and exclusion criteria

The identified articles were uploaded to Rayyan, a web platform for systematic reviews [35]. Four independent investigators (KW, JM, EA, and KK) reviewed and extracted the data, and disagreements were resolved through discussion and consultation with LIOO. Studies were included if they were conducted in Kenya, were published in English, and focused on molecular markers of resistance in *Plasmodium falciparum*. Studies were excluded if they addressed pathogens other than *P. falciparum,* were purely therapeutic efficacy trials or targeted populations beyond the geographical limits of Kenya. Furthermore, studies that did not provide genotype frequencies or consisted solely of reviews of genotyping studies were also excluded. Full-text articles that were not readily accessible were obtained with the librarian's assistance at the KEMRI-Wellcome Trust Research Programme.

### Screening and data extraction

The 110 studies that met the inclusion criteria were systematically reviewed, and data were extracted into comma-separated files (CSV) for each study based on predefined variables (Table 1). A set of R scripts was used to manage and structure the extracted data. Raw allele and microhaplotype frequency data were imported and cleaned. This included parsing fields to extract codon positions, distinguishing wildtype from mutant alleles, and appending standardized gene identifiers from PlasmoDB [36, 37]. The cleaned allele and microhaplotype tables were saved for downstream use. Aggregated data from the publications was utilized to generate summary plots of mutation and microhaplotype frequency data by malaria epidemiology zones (Fig. 1) and year. Frequencies were either extracted directly or calculated from reported counts, and plots of data availability and temporal trends were generated to guide interpretation. All the extracted variables were compiled into master tables summarizing allele and microhaplotype prevalence, resistance profiles and spatial–temporal trends. These data were synthesized to map the distribution of drug resistance markers across Kenya and to identify key gaps in current surveillance.

**Table 1:** List of variables extracted from the 110 studies

Variable
PMID
Year of publication
Study county
Study town
Year of sample collection
Number of samples genotyped (sample size)
Number of samples genotyped
Target gene
Target allele
Number of wildtype genotypes
Number of mutant genotypes
Number of mixed genotypes
Codons
Microhaplotype
Microhaplotype frequency
Genotyping assay
Participants age
Participants age unit (years or months)
Participants clinical status (asymptomatic, symptomatic or severe)
Study design (health facility-based or community survey)
Data extraction comments

### Resistance marker classification

To support interpretation of spatiotemporal patterns in antimalarial resistance, a reference list of key molecular genetic markers (*crt*, *dhfr*, *dhps*, *k13*, *mdr1*) associated with reduced sensitivity to three major drug classes (CQ, SP, and ACT) was compiled (Table 2). These markers (their associated codons or mutations) form the foundation of molecular surveillance efforts and are critical for assessing the emergence and spread of resistance, guiding treatment policy, and supporting early warning systems. Other putative drug resistance markers were included based on literature providing evidence of their potential role in conferring resistance mainly to ACT. Other markers such as *ap2-mu, ubp-1*, *exo* and *coronin*, though less well-characterized, are included for completeness, as they have been reported in local studies and may represent early indicators of emerging resistance. This classification framework supports the rationale for gene inclusion in this review and provides a clear reference point for interpreting trends in resistance across time, regions, and drug classes.

**Table 2:** Summarizes the most frequently studied resistance molecular markers in malaria-endemic regions

Gene	Codon(s)/mutation(s)	Drug class	Role in resistance	References
*crt*	M74I, N75E, K76T, H97Q, A220S, Q271E, N326S, I356T, C350S, R371I	Chloroquine (CQ)	Key mutations conferring CQ resistance	[47, 57, 58]
*mdr1*	N86Y, Y184F, S1034C, N1042D, D1246Y and copy number variation (CNV)	CQ, Amodiaquine (AQ), Mefloquine (MQ), ACT partner drugs	Modulates CQ and MQ response, lumefantrine tolerance	[59]
*dhfr*	N51I, C59R, S108N, I164L	Pyrimethamine (SP)	Core resistance to pyrimethamine	[60–62]
*dhps*	S436A, A437G, K540E, A581G, A613S	Sulfadoxine (SP)	Core resistance to sulfadoxine	[63]
*k13*	Mutations in the propeller domain	Artemisinin (ACT)	Validated markers of partial resistance	[64]
*plasmepsin-II/III*	CNVs	Piperaquine (ACT)	Associated with treatment failure	[65, 66]
*coronin*	R100K, G50E	ACT	Reduced susceptibility (lab evidence)	[67]
*mrp1*	I876V	ACT background	Alters drug efflux	[68]
*ap2-mu*	S160N/T	Artemisinin (ACT)	Linked to linked to delayed clearance after ACT	[69]
*ubp1*	E1528D, ﻿V3275F	ACT background	Modulates artemisinin response	[69, 70]
*exo*	E415G	Piperaquine (ACT)	Linked to piperaquine failure in Asia	[71]
*atp6*	E431K, A623E, S769N	Artemisinin (ACT)	Modulates artemisinin response	[72, 73]
*falcipain-2a*	S69Stop	Artemisinin (ACT)	Modulates artemisinin response	[74]
*nfs*	K65Q	Lumefantrine (ACT)	Lumefantrine tolerance	[75]
*arps10*	V127M	Artemisinin (ACT)	Artemisinin background mutations	[76]
*mdr2*	T484I	Artemisinin (ACT)	Artemisinin background mutations	[76]
*fd*	N193Y	Artemisinin (ACT)	Artemisinin background mutations	[76]

## Results

### Basic characteristics of included studies

**Fig. 2:**
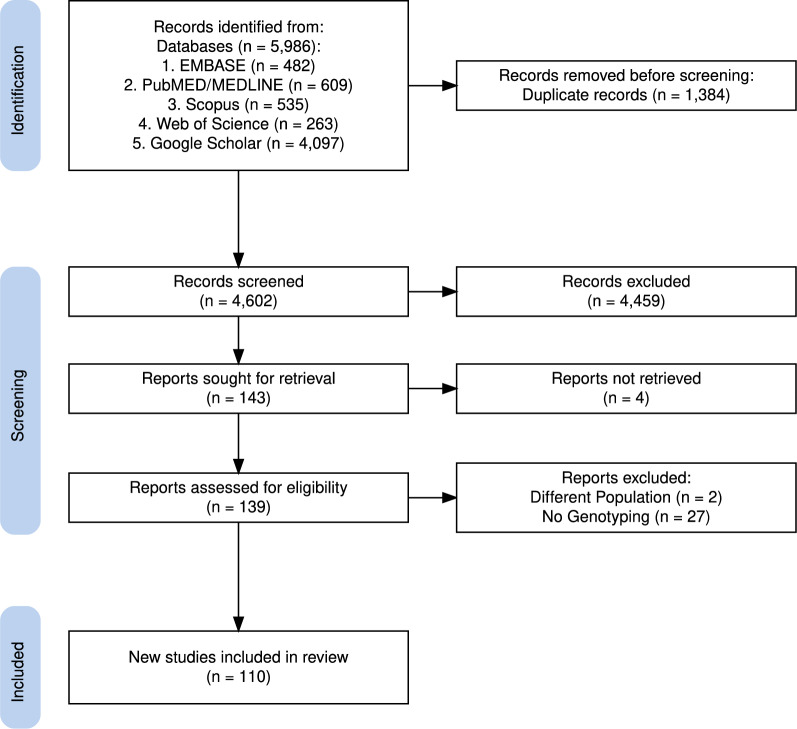
PRISMA Flow Diagram of Study Selection. The figure illustrates the study selection process following PRISMA guidelines. After removing duplicate records, the remaining studies underwent screening based on titles, abstracts, and full texts. Studies that did not meet the eligibility criteria, including those without genotyping data or focusing on non-*Plasmodium falciparum* pathogens, were excluded. The final number of studies included in the review was 110. This figure was generated using the PRISMA2020 R package v.1.1.3 [56]

5986 reports meeting the broad search criteria were identified from the database searches, there were 1384 duplicate records across the searched databases that were removed. The remaining 4602 titles and abstracts were screened, resulting in the exclusion of 4460 records not meeting the scoping review’s inclusion criteria (Fig. 2). Of the remaining publications four could not be retrieved. Therefore, the full texts of 138 studies were assessed for eligibility, 29 studies were excluded (2 were from populations outside Kenya and 27 did include genotyping data). 110 studies were included in the review (Fig. 2). Studies included were undertaken between 1987 and 2024, with > 70% (646/923) being undertaken between 2005 and 2019 (Table 2). Participants across all studies were aged between 1 month to 85 years. 70.8% of reported studies focused on uncomplicated malaria, followed by 13.8% asymptomatic and 1% severe malaria, while 2.1% of cases were not reported. Over two-thirds (62.1%) of the studies were clinic-based, with 11.3% assessing treatment response in therapeutic efficacy studies. Community surveys comprised 20%, while 5.6% had unspecified study designs due to missing data (Table 3).

**Table 3:** Demographic characteristics of study participants

	Central (N = 2)	Coast (N = 78)	Eastern (N = 1)	Rift Valley (N = 11)	Western (N = 88)	Multiple (N = 14)	Unknown (N = 1)	Overall (N = 195)
Age group								
≤ 5	1 (50.0%)	17 (21.8%)	0	4 (36.4%)	34 (38.6%)	1 (7.1%)	1 (100%)	58 (29.7%)
≤ 15	0	23 (29.5%)	1 (100%)	0 (0%)	12 (13.6%)	0	0	36 (18.5%)
> 15	1 (50.0%)	9 (11.5%)	0	5 (45.5%)	14 (15.9%)	12 (85.7%)	0	41 (21.0%)
*Missing*	0	29 (37.2%)	0	2 (18.2%)	28 (31.8%)	1 (7.1%)	0	60 (30.8%)
Participants clinical case								
Uncomplicated	2 (100%)	52 (66.7%)	0	9 (81.8%)	61 (69.3%)	13 (92.9%)	1 (100%)	138 (70.8%)
Asymptomatic	0	3 (3.8%)	0	1 (9.1%)	22 (25.0%)	1 (7.1%)	0	27 (13.8%)
Asymptomatic,uncomplicated*	0	23 (29.5%)	0	0 (0%)	1 (1.1%)	0	0	24 (12.3%)
Severe	0	0	1 (100%)	0 (0%)	1 (1.1%)	0	0	2 (1.0%)
*Missing*	0	0	0	1 (9.1%)	3 (3.4%)	0	0	4 (2.1%)
Study design								
Clinic	2 (100%)	50 (64.1%)	1 (100%)	8 (72.7%)	52 (59.1%)	7 (50.0%)	1 (100%)	121 (62.1%)
Clinic/community survey	0	23 (29.5%)	0	0	0	0	0	23 (11.8%)
Community survey	0	4 (5.1%)	0	2 (18.2%)	29 (33.0%)	4 (28.6%)	0	39 (20.0%)
Vaccine trial (clinic)/community survey*	0	1 (1.3%)	0	0	0	0	0	1 (0.5%)
*Missing*	0	0	0	1 (9.1%)	7 (8.0%)	3 (21.4%)	0	11 (5.6%)

‘*’ indicates studies that collected samples across multiple classification categories, either multiple clinical case definitions or multiple study design types; ‘multiple’ refers to the studies that include data from an aggregate of counties across different regions of the country; ‘missing’ indicates data not available in the study and ‘unknown’ denotes that the study did not provide detail on county or region

Across Kenya's five malaria epidemiological zones (Fig. 1) there were significant variations in data density over the review period. Lakeside and coastal endemic zones characterized by historically high and moderate-to-high malaria transmission had the most comprehensive published molecular surveillance data. Data from the Highland epidemic-prone zones was also present, particularly for the historically important markers, reflecting efforts to monitor areas susceptible to malaria outbreaks (Table 4). Conversely, molecular data were notably sparse and more fragmented in the semi-arid seasonal transmission zones and the low-risk areas (Table 4).

**Table 4:**
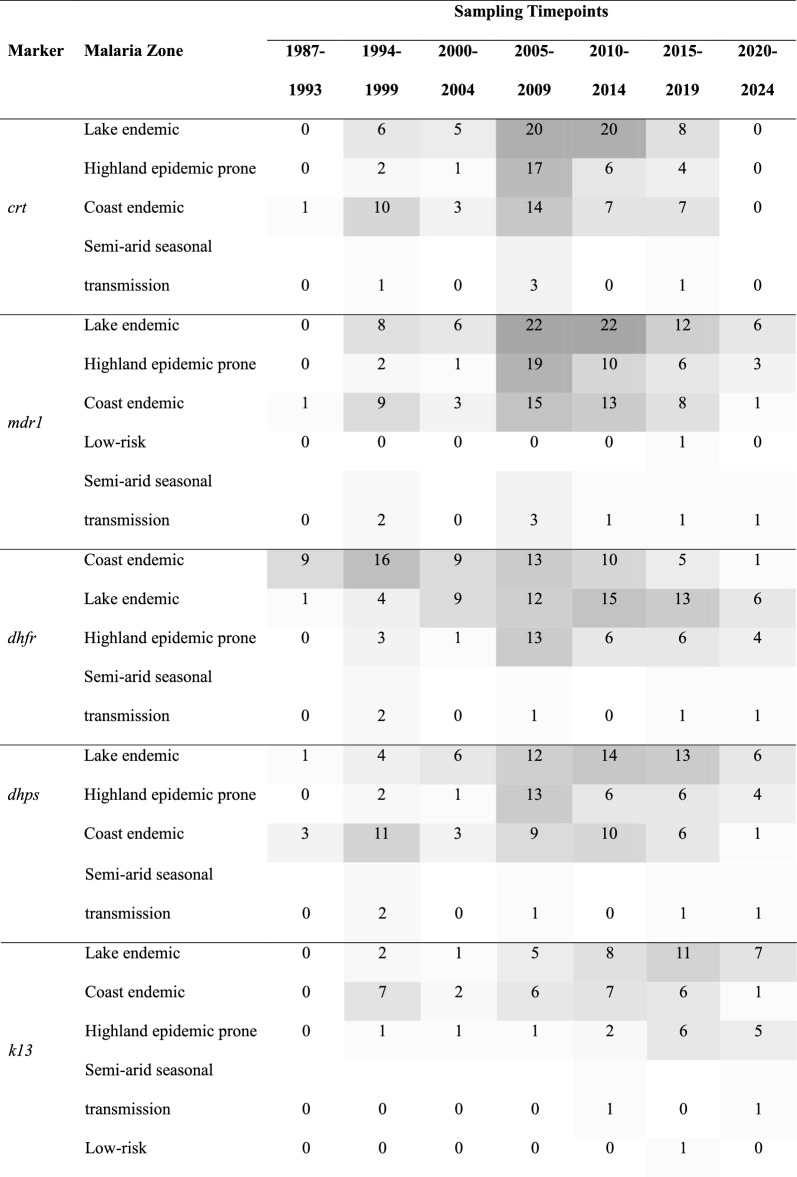

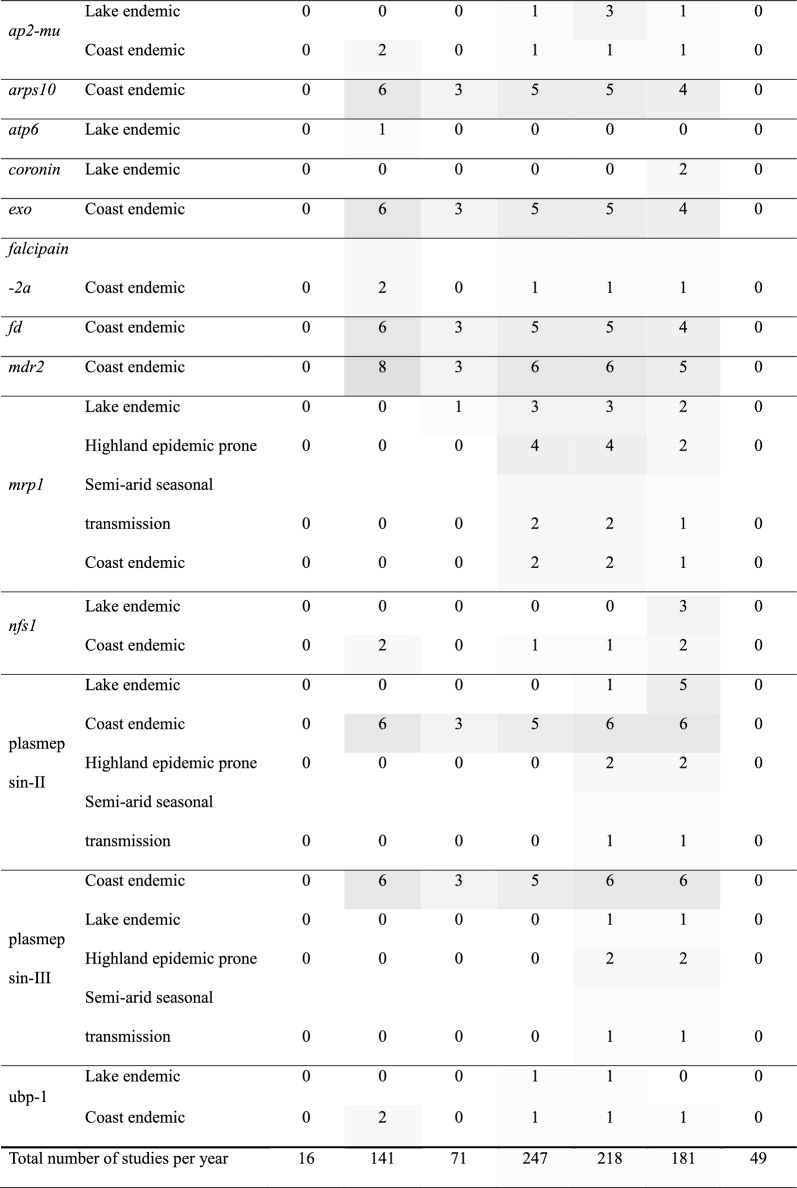
Number of studies sampling across time periods and malaria zones in Kenya. The darker the grey shade the higher the frequency

### Molecular surveillance methodologies timeline

The early years (1997–2005) were dominated by Restriction Fragment Length Polymorphism (RFLP) and Sanger sequencing. By 2006, real-time quantitative PCR (qPCR) emerged and maintained consistent usage. From 2012 onwards, next-generation sequencing (NGS) methods, such as amplicon and whole-genome sequencing (WGS), were more prevalent, while SNP genotyping, the MassArray technology, gained traction after 2015, with increased use from 2020 to 2025. More niche applications, Sequence-Specific Oligonucleotide Probe (SSOP) hybridization assays, SSOP-ELISA, and yeast-expression systems appeared intermittently.

### Nationwide molecular marker surveillance profiles

**Fig. 3:**
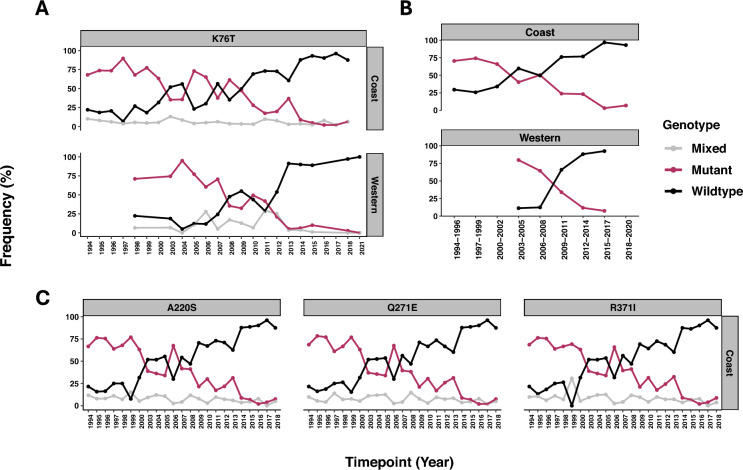
Temporal trends in the frequency of Pfcrt mutations from 1994 to 2018. The **A** K76T; (**C**) A220S, Q271E, and R371I genotypes; and **B** microhaplotypes CVIET (mutant) and CVMNK (wildtype) are shown. The data was stratified by region: Coast (top row) and Western Kenya (bottom row), for where data was available for both regions. **C** did not have data from Western Kenya for codons 220, 271 and 371. There was a progressive increase in the frequency of wildtype microhaplotype and alleles were observed at all 4 loci over time

The data together corroborates the previously published work, with the declining *crt* 76 T resistance conferring mutation, and both the Coast and Western Kenya showing a complete reversion to the wildtype, CVMNK genotype, by 2022 (Fig. 3A). This is the exact same pattern for resistant mutant amino acids at other genetic loci within the crt gene codons 220, 271 and 371 (Fig. 3B). The national analysis shows a regional shift in the timelines for the mutant to wild-type *crt* genotype switches and similarly for the mutant (CVIET) to wild-type (CVMNK) microhaplotype switch (Fig. 3C), with the Coast occurring in 2002, while Western Kenya occurred later in 2008.

**Fig. 4:**
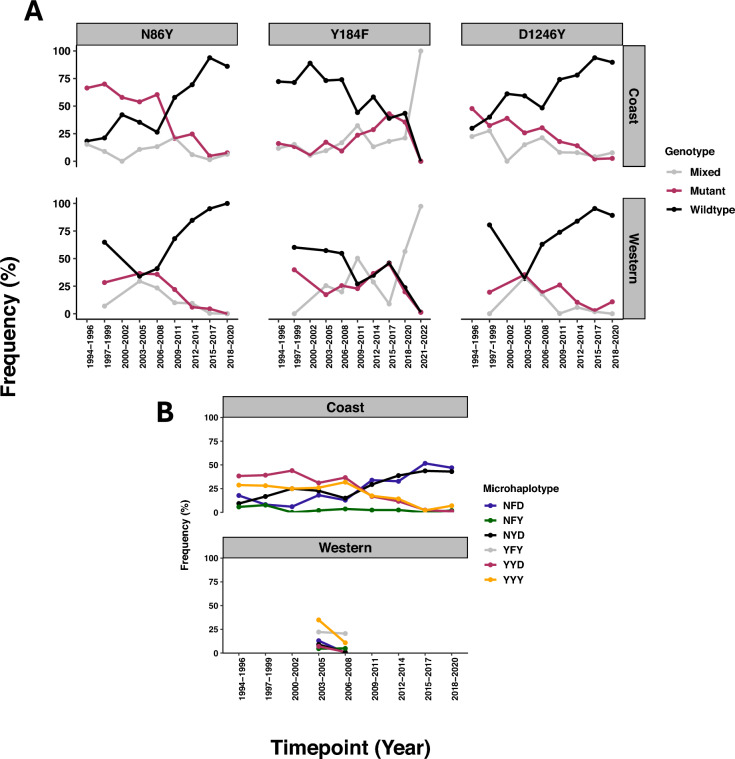
Temporal trends in the frequency of *Pfmdr1* genotypes in Coastal and Western Kenya regions from 1994 to 2022. **A** Each panel shows the proportions of wildtype (black), mutant (red), and mixed (grey) genotypes at three codon positions in Pfmdr1 gene over time. A notable spike in mixed Y184F genotypes during 2021–2022 in Western Kenya (100%) resulted from the fact that only one study [40] contributed data during this timepoint, and majority of the samples from that dataset were mixed infections. No data was available for codons N86Y and D1246Y beyond 2020. **B** The NFD microhaplotype, associated with reduced lumefantrine susceptibility, has shown a steady increase in Coastal Kenya at similar proportions to the wildtype, NYD microhaplotype. The triple mutant YFY was only detected at low frequencies in Western Kenya between 2003 and 2008. Other haplotypes, including NFY, YYD, and YYY, remained at low prevalence over time

Furthermore, MDR1 codons 86 and 1246, also genetic markers of chloroquine resistance have shown a full reversion to the wildtype, that was rising since 1994 in the Coast and 2003 in Western Kenya. In contrast, the codon 184 wildtype started dropping in 2006. The NFY and NYD haplotypes together rise in frequency from 2006 and the rare YFY (184 and 1246 double mutant) is only observed in Western Kenya (Fig. 4).

**Fig. 5:**
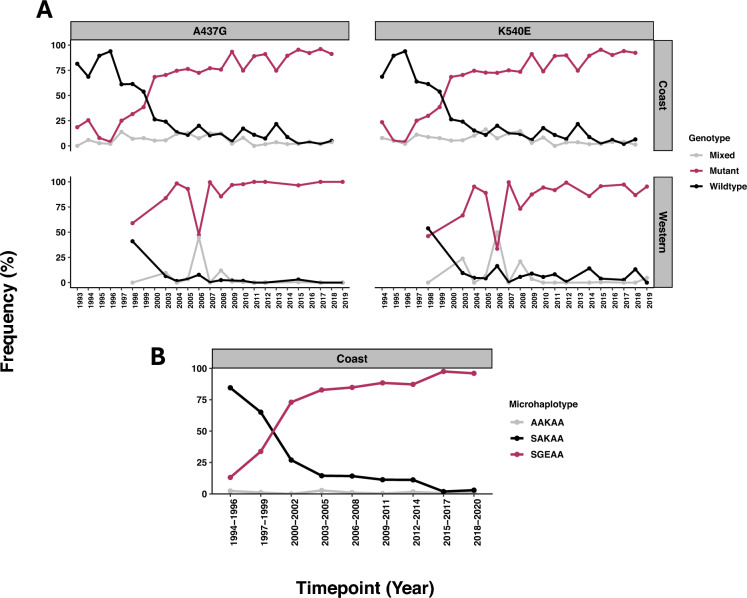
The temporal trends in the dhps alleles, marker for sulfadoxine resistance. The mutant 437G and 540E genotypes remained near fixation from 2016. However, a notable dip in their frequencies was observed in 2006, primarily due to a high proportion of mixed genotypes reported at that timepoint. The dhps SGEA double mutant microhaplotype, characterized by the alleles at codons S436, 437G, 540E, and A581, steadily increased over time in the coastal region, reaching near fixation by 2015. In contrast, the wild-type SAKA haplotype declined sharply over time to 0% also by 2015. A remarkable shift between these haplotypes took place between 1999 and 2000, coinciding with the replacement of chloroquine with sulfadoxine-pyrimethamine

For *dhps*, by the time drug policy changed to SP in 1999 the mutant was already rising from 1996 in the Coast and 1998 from Western Kenya (based on available data) (Fig. 5A). The mutant genotype was already taking over as the dominant genotype in both regions of the country, clearly demonstrated in the dhps haplotype analysis from 1994 with a wild type to mutant shift by 1999 (Fig. 5B). Of note, across both regions of the country codons 436, 581 and 613 were predominantly wildtype over time.

**Fig. 6:**
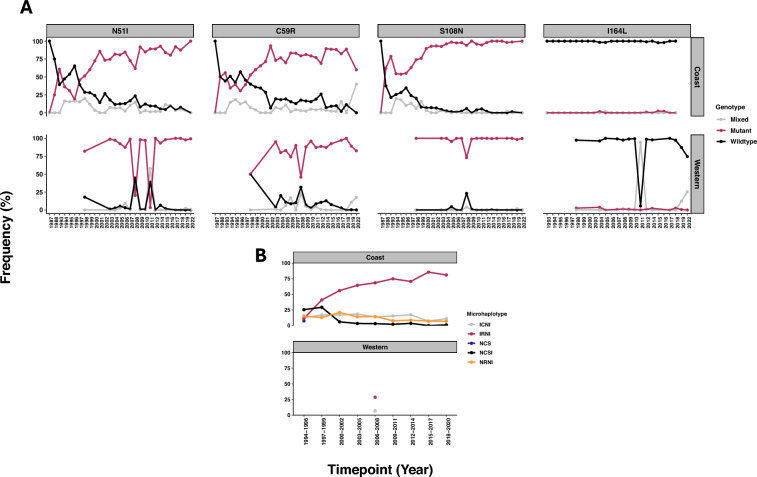
Temporal trends in dhfr alleles across Coastal and Western Kenya regions. **A** The switch from wildtype to mutant alleles occurred as early as 1998 for 3 loci, 51, 59 and 108. Codons 51I and 108N mutations were near fixation in both regions of the country. Due to incomplete data, the 2013 timepoint for Western Kenya was excluded from the plot but is available in Supplementary Table 1. Furthermore, in Western Kenya, some studies reported a high number of mixed infections that altered the temporal trend, highlighting the need for consistency in defining mutations across studies and time. The codon 164L mutation is beginning to emerge in mixed infections in Western Kenya revealing the reduction in wildtype parasites, warranting continued surveillance. **B** The temporal trends in dhfr microhaplotypes. In Coastal Kenya, a distinct shift from the wildtype NCSI to the triple mutant IRNI microhaplotype occurred from 1996. The IRNI has steadily increased in frequency, approaching fixation in the later time periods. The wildtype is rare and mostly absent since 2003 along the Coast. In Western Kenya, temporal trends could not be assessed due to missing data for several key years

For the pyrimethamine resistance marker, *dhfr*, as early as 1988, codon 108 started to shift to the mutant genotype in the Coastal parasite populations, codons 51 and 59 shifted later in 1996 (Fig. 6A), also reflected in the triple mutant IRNI haplotype in the Coast (Fig. 6B). The Western Kenya data was patchy and thus an assessment of the earliest time points the mutations rose in frequency could not be made and there was no microhaplotype data apart from 2006 to 2008. Furthermore, there has a high reporting of mixed infections in 2008 compared to all other time points, resulting in a drastic drop in the mutant frequencies in Western Kenya. From 2018, the wildtype amino acid at codon 164 showed a decline in frequency in Western Kenya only, where genotyping data is available post-2018 (Fig. 6A).

A common *k13* mutation across time was the A578S but at a low frequency (< 5%). Emerging WHO validated *k13* mutations were first described, P553L, in 2006 in Kisumu at 2% (Table 5). However, since 2022 the observations of the validated mutations, C469Y, P553L and A675V, was geographically widespread across Western Kenya. There were no observations of these mutations along the Coast, apart from a frequent observation of a synonymous change at codon C469C from 1998 in Kilifi and observations across counties in Western Kenya to 2022 and a single observation of R539K in 2013 in Malindi (Supplementary Table 2). There was a WHO mutation associated with resistance, V568G in 2003 and 2013 in Kisumu at low frequency 5.4% and 2.4%, respectively. Similarly, S552C was observed in 1994 in Kilifi, another WHO mutation associated with resistance (Supplementary Table 2).

**Table 5:** List of WHO validated K13 mutations in Kenya

Sampling year	County	Malaria epidemiological zones	Sample size	N458Y	C469Y	R539T	P553L	R561H	A675V	References
2006	Kisumu	Lake endemic	50	–	–	–	2	–	–	[42]
2018–2022	Kisumu, Kisii, Kakamega, Homa Bay	Lake and highland epidemic	775	–	–	–	–	–	0.4	[38]
2021	Kisii	Highland epidemic	13	20	–	20	–	40	–	[41]
2022	Bungoma	Highland epidemic	74	–	5.9*	–	–	–	–	[40]
2022	Busia	Lake endemic	322	–	2.8*	–	–	–	–	[40]
2022	Busia	Lake endemic	226	–	4.8	–	–	–	–	[39]
2022	Kakamega	Highland epidemic	145	–	–	–	4.3*	–	–	[40]
2022	Kisumu	Lake endemic	161	–	10.7*	–	3.6*	–	7.1*	[40]
2022	Migori	Lake endemic	275	–	2.1*	–	–	–	–	[40]
2022	Siaya	Lake endemic	337	–	6.9*	–	–	–	–	[40]
2022	Turkana	Seasonal transmission	92	–	4.3*	–	–	–	–	[40]
2022	Vihiga	Lake endemic	68	–	2.1*	–	–	–	–	[40]
2022	West Pokot	Seasonal transmission	32	–	33.3*	–	14.3*	–	50*	[40]

An asterisk (*) denotes mixed genotype frequencies, where both wild-type and mutant alleles were detected within the same sample

Dash (–) indicates that the mutation was not detected or not assessed in the study

There was data on other putative drug resistance markers (based on previous publications, Table 1, Supplementary Table 3) that showed no change in allele frequency trends over time (*ap2mu, atp6, falcipain-2a, mrp1, nfs* and *ubp1*) and those that were 100% wildtype (arps10, exo, fd and mdr2). However, for coronin, only one study reported data over a two-year period; therefore, no trend analysis was performed. The data is available in Supplementary Table 1.

## Discussion

This scoping review identified data variables that can be utilized to generate a shared data standard for the country. It can be populated as an aggregate dataset that provides a nationwide overview of the presence and frequency of mutations over time. Of immediate importance is the continued presence of WHO-validated *k13* mutations in Western Kenya, from Turkana southwards to Homa Bay and Migori counties [38–40]. Due to the low numbers of samples (and reported high frequency) from Maniga and colleagues [41], the sequence data available on GenBank was reassembled to countercheck the reported frequencies, and the sequence alignment analysis was not able to verify the presence of the reported SNPs. However, this review highlights the early identification of a now WHO-validated mutation, P553L, in a study published in 2006 [42]. It also points to the detection, as early as 1998 [43], of a synonymous change (C469C) at a locus that has since been validated by the WHO, where the clinically relevant C469Y mutation later emerged. Both are important additions towards understanding the timelines of genetic changes at the *k13* locus nationally.

Other important potentially emerging mutations that require continued monitoring are the *dhfr* 164L mutation that will make the parasites super-resistant to SP and for which data is required from the Coast; and the *dhps* 581G mutation, known to be associated or to arise due to intermittent preventive treatment in pregnancy (IPTp) [44], that is still wildtype. The *dhfr* and *dhps* genes should continue to be tracked in Western Kenya, given the limited data in this review, where the malaria burden is highest and SP in IPTp is a major malaria control measure. The early shift to the mutant genotypes for *dhps* and *dhfr* reflects directional selection pressure exerted by widespread SP use, driving the expansion of the resistant parasite populations.

CQ has reverted to a > 99.9% wild type population across Kenya. The delays in resistance switches between the East and West of the country highlight the potential lack of uniformity in the complete withdrawal of chloroquine in the late 90 s during the national policy change to SP. These are lessons that are useful for any future drug regimen changes and an important indicator to work closely with the private sector on national treatment changes.The *crt* gene and variant (76 T) is the primary mediator of CQR, increasing the export of CQ from the food vacuole away from its target haem [45]. Additionally, MDR1 modulates the parasites to several antimalarial drugs, including CQ, it is involved in the hydrophobic antimalarial efflux [46]. Additional mutations following the *crt* temporal pattern, codons 220, 271 and 371, were described in the Coastal dataset and is missing from Western Kenya, a gap that requires data. These loci were identified by whole genome sequencing, showing the value of NGS to identify additional loci. The current complement of 7 *crt* point mutations along the Coast are from codons 72, 74, 75, 76, 220, 271 and 371, which is consistent with the pattern for parasites from Africa that also includes codon 236 [47] that was wildtype. Furthermore, the South American combination includes codon 356 [47], which was also wild-type in the current dataset. The two *Pfmdr1* mutations 86Y and 1246Y (found in Africa) mediate the parasite’s decreased susceptibility to chloroquine and amodiaquine, but increased sensitivity to lumefantrine, mefloquine, and artemisinins [48–50]. These mutations followed the K76T pattern of a reduction of the mutation alleles over time. Additional mutations 1034C and 1042D (observed outside Africa) have been associated with altered sensitivity to lumefantrine, mefloquine, and artemisinins [49, 51–53]. These mutations were not observed in Kenya as these loci were primarily wildtype.

Based on the search terms examined across malaria-endemic regions in Kenya, the majority of long-term data is concentrated in the Coast and Western regions. This is not unexpected, as these areas host national research partners with sustained investment in research infrastructure and international collaborations (e.g., KEMRI collaborative centres in Kisumu and Kilifi). Nevertheless, there remains a need for more data from other malaria-endemic regions to ensure a more comprehensive national picture. The semi-arid seasonal transmission zones and the low-risk areas have traditionally been neglected by the malaria research communities in Kenya, due to the low infection rates and disease burden and despite these regions having frequent malaria outbreaks. These geographical gaps in data are important to redress through future surveillance efforts as the epidemiology of mutation rates could be different in low transmission, semi-arid, pastoralist areas bordering other countries for a more comprehensive national data repository.

This aggregation of data across Kenya demonstrates the utility of this scoping review. In isolation, based on the focus of the research institutions working in Western Kenya and along the Coast, the molecular data corroborated the national changes in antimalarial drug policy. Furthermore, it demonstrates the power of a consolidated database and the essential variables to support data sharing (Table 1). The compilation and standardization of over 100 studies provides a high-level, structured overview of when and where resistance markers have been surveyed. This establishes a foundational national repository to support strategic surveillance planning by the Kenya NMCP. It further highlights the urgent need for more equitable surveillance efforts to ensure national representation. A centralized MMS repository will allow for the necessary resources and technical support to be mobilized; expertise to be shared by developing a network of laboratories for genomics and bioinformatics; standardized protocols to generate reproducible data and enable reagents sourcing at scale; and importantly coordinated sample referral systems [54]. For malaria this is important as control interventions can be targeted to regions where resistance is emerging as evidenced from the current data in Western Kenya that also highlights the need for border control interventions. The purpose of this review is not to claim complete national coverage but to serve as a structured repository of all available data while highlighting critical gaps. For the NMCP, its value lies in guiding the prioritization of new surveillance investments in underrepresented regions, identifying emerging hotspots (such as verified *k13* mutations in Western Kenya), and providing insights from well-studied areas to inform national policy. In line with this, a policy brief has been shared with the NMCP, while a complementary technical report has been developed for researchers in the country to populate in near real-time, thereby strengthening the foundation for a nationally representative surveillance framework.

## Limitations

This review has several limitations. First, as a scoping review, this study did not formally appraise the quality of included studies, resulting in data from methodologically weaker studies being treated equally with data from stronger studies. Second, unlike a systematic review with meta-analysis, this study’s approach does not generate pooled prevalence estimates; instead, it provides a descriptive mapping of available evidence, which limits the precision of national-level estimates. Third, these findings rely on published studies only, raising the possibility of publication bias, as unpublished or negative findings may be underrepresented. Fourth, there was variability across studies in genotyping methods and in how “mixed” versus “mutant” infections were defined, which makes direct comparisons between studies challenging and may partly explain differences observed over time or by region. Mixed infections may sometimes be referred to as mutants since they are likely to lead to a resistance phenotype. Fifth, there remains a geographical imbalance. Although data from malaria-endemic regions across Kenya were examined, most long-term data are concentrated in the Coast and Western regions, where research institutions and international collaborations are based. This means that other malaria-endemic regions, including semi-arid seasonal transmission zones and low-risk outbreak-prone areas, remain underrepresented despite their importance. Finally, Shifts in molecular methodology were noted, transitioning from traditional approaches to advanced sequencing technologies. This variability underscores the need for standardized definitions (e.g., for mixed infections), as well as harmonized laboratory protocols, analytical pipelines, and reporting standards to ensure reproducibility and comparability. Taken together, these limitations mean that these findings should be interpreted as a structured overview of existing knowledge and evidence gaps, rather than precise national estimates.

## Supplementary Information

Additional file 1.

Additional file 2.

Additional file 3.

## Data Availability

All data generated or analysed during this study are included in this published article and its supplementary information files.
